# General Patterns and Species-Specific Differences in the Organization of the Tubulin Cytoskeleton in Indeterminate Nodules of Three Legumes

**DOI:** 10.3390/cells10051012

**Published:** 2021-04-25

**Authors:** Anna B. Kitaeva, Artemii P. Gorshkov, Evgenii A. Kirichek, Pyotr G. Kusakin, Anna V. Tsyganova, Viktor E. Tsyganov

**Affiliations:** Laboratory of Molecular and Cellular Biology, All-Russia Research Institute for Agricultural Microbiology, Podbelsky Chaussee 3, Pushkin 8, 196608 Saint-Petersburg, Russia; anykitaeva@gmail.com (A.B.K.); artemius1993@yandex.ru (A.P.G.); jenykir@rambler.ru (E.A.K.); kussakin@gmail.com (P.G.K.); avtsyganova@arriam.ru (A.V.T.)

**Keywords:** legume–rhizobial symbiosis, microtubules, symbiosome, bacteroid, indeterminate nodules, immunolocalization, *Vicia sativa*, *Galega orientalis*, *Cicer arietinum*

## Abstract

The tubulin cytoskeleton plays an important role in establishing legume–rhizobial symbiosis at all stages of its development. Previously, tubulin cytoskeleton organization was studied in detail in the indeterminate nodules of two legume species, *Pisum sativum* and *Medicago truncatula*. General as well as species-specific patterns were revealed. To further the understanding of the formation of general and species-specific microtubule patterns in indeterminate nodules, the tubulin cytoskeleton organization was studied in three legume species (*Vicia sativa*, *Galega orientalis*, and *Cicer arietinum*). It is shown that these species differ in the shape and size of rhizobial cells (bacteroids). Immunolocalization of microtubules revealed the universality of cortical and endoplasmic microtubule organization in the meristematic cells, infected cells of the infection zone, and uninfected cells in nodules of the three species. However, there are differences in the endoplasmic microtubule organization in nitrogen-fixing cells among the species, as confirmed by quantitative analysis. It appears that the differences are linked to bacteroid morphology (both shape and size).

## 1. Introduction

The symbiotic interactions between legumes and rhizobia culminate in the formation of nitrogen-fixing root nodules [1]. In response to flavonoids produced by legume roots, rhizobia secrete specific lipochitooligosaccharides, known as Nod factors, which trigger nodule development [2]. In legumes that form indeterminate nodules with a persistent meristem, nodule formation starts with root hair curling and the formation of an infection thread [3]. Simultaneously, cell division is reactivated in the pericycle and inner cortex, leading to nodule primordium formation [4,5]. When an infection thread reaches a primordium, an infection droplet lacking a cell wall is formed. The bacteria are then released into the host cell cytoplasm but they remain surrounded by a plasmalemma [6], forming an organelle-like symbiosome [7]. Indeterminate nodule development is accompanied by the cell differentiation of both symbiotic partners [7]. The plant cells significantly increase in volume after several rounds of endoreduplication, which increases the DNA content to the 64C stage [8]. The significant increase in the volume of a nodule cell allows it to host up to 50,000 differentiated rhizobial cells (bacteroids) [9]. Concurrently, some plant cells remain uninfected [6]. The bacteroids in legumes in the inverted repeat-lacking clade (IRLC) undergo terminal differentiation, which is triggered by nodule-specific cysteine-rich (NCR) peptides [10].

The tubulin cytoskeleton plays a significant role in nodule development [11,12]. In growing root hairs, cortical microtubules are mainly oriented parallel to the growth axis and do not reach the tip of the root hair [13]. Endoplasmic microtubules link the nucleus to the tip of the root hair, forming a dynamic pattern [13,14]. Treatment of *Medicago truncatula* Geartn. root hairs with Nod factors decreased the cortical and endoplasmic microtubule dynamics and the microtubule growth rate [13]. In *Lotus japonicus* (Regel) K. Larsen, the addition of Nod factors also decreased the microtubule growth rate [15].

Microtubular organization at the early stages of nodule development was studied during the interactions of *Sinorhizobium meliloti* with *Medicago sativa* L. and *M. truncatula* [4]. In curled root hairs, endoplasmic microtubules are associated with the site of infection. The tip of the growing infection thread is linked to the migrating nucleus via endoplasmic microtubules. Longitudinal endoplasmic microtubules are located along the infection thread. As cell division in the outer cortex begins, the microtubules are rearranged. As a result, the endoplasmic microtubules are oriented parallel in the cytoplasmic bridge, which connects different sides of the cell and forms a pre-infection thread; the cortical microtubules disappear. Microtubule reorganization also occurs in the pericycle. In the cells of the inner cortex, cortical microtubules change their orientation from a regular pattern (oriented parallel to each other) to an irregular one. Endoplasmic microtubules link the nucleus to the cell periphery [4]. Nevertheless, the molecular mechanisms underlying the reorganization of the tubulin cytoskeleton at the early stages of symbiotic nodule development have rarely been studied. Only recently *M. truncatula* DEVELOPMENTALLY REGULATED PLASMA MEMBRANE POLYPEPTIDE (DREPP), which is involved in early root hair responses to rhizobia, was identified [16]. However, this is still the only example of the identification of proteins that control the organization of microtubules during nodule development.

The tubulin cytoskeleton in mature symbiotic nodules has also been studied in *Pisum sativum* L. [17], *Lupinus albus* L. [18], *Glycine max* (L.) Merr. [19], *Macroptilium atropurpureum* (DC.) Urb., and *M. truncatula* [20]. In these studies, the involvement of the tubulin cytoskeleton during the bacterial infection of nodule cells was explored. It was assumed that the tubulin cytoskeleton is involved in the distribution of symbiosomes and organelles. The natural or induced senescence of nodules in *P. sativum* is accompanied by microtubule depolymerization [21].

Recently, a detailed analysis of tubulin cytoskeleton organization was performed in cells of mature nodules of *P. sativum* and *M. truncatula* [22]. It was shown that endoplasmic microtubules create a matrix for the growth of an infection thread, support infection droplets, and are involved in the distribution of symbiosomes in infected cells. Cortical microtubules were distributed in a regular pattern in uninfected and colonized cells and an irregular pattern in infected cells. Additionally, there were differences in the endoplasmic microtubule patterns between the *P. sativum* and *M. truncatula* nodules, and it was suggested that these differences were caused by differences in the morphology of the bacteroids [22]. Legumes differ considerably in the morphology of bacteroids. At present, three main morphotypes of bacteroids are recognized: (1) E morphotype, i.e., elongated (subdivided into branched (B) and not branched (NB)); (2) S morphotype, i.e., swollen and spherical; and (3) U morphotype, i.e., no increase in bacteroid size and the rod-like shape characteristic of free-living rhizobia is maintained [23,24]. The morphology of bacteroids can be an important feature, as a correlation was found between the morphology of bacteroids and the efficiency of nitrogen fixation. The higher symbiotic efficiency was demonstrated for E and S morphotypes in comparison with U [25,26]. The symbiotic efficiency of the highly polyploid S morphotype was higher than for the low polyploid E morphotype in *Aeschynomene* spp. [27]. Likely, the distribution of symbiosomes in a cell can also affect their efficiency. Therefore, the identification of the mechanisms of distribution of symbiosomes in a cell is an important task. Tubulin cytoskeletons are important elements of these mechanisms.

This study aimed to analyze the cortical and endoplasmic microtubule organization in the indeterminate nodules of three legume plant species (*Vicia sativa* L., *Galega orientalis* Lam, and *Cicer arietinum* L.) to identify general and species-specific patterns of tubulin cytoskeleton organization and to try to link the species-specific patterns to the bacteroid morphology. The bacteroids of *V. sativa* and *G. orientalis* belong to the EB morphotype [28,29], whereas the bacteroids of *C. arietinum* belong to the ENB [29] and S [30] morphotypes.

## 2. Materials and Methods

### 2.1. Plant Material and Bacterial Strains

Commercial seeds of *V. sativa* cultivar Lgovskaya 22, *G. orientalis* cv. Gale, and *C. arietinum* cv. Chickpea of Spello were used. The *C. arietinum* seeds were sterilized in 96% ethanol for 10 min; the *G. orientalis* and *V. sativa* seeds were sterilized in concentrated sulfuric acid for 5 and 8 min, respectively. After sterilization, the seeds were washed with sterile water 10 times. The seedlings were then inoculated with 1 mL of an aqueous suspension of one of the following rhizobial strains (10^7^–10^8^ cells/seedling): *Rhizobium leguminosarum* bv. *viciae* Vs 35-4 (*V. sativa*), *Rhizobium galegae* Gr 32 (*G. orientalis*) (both obtained from the Collection of Laboratory of Microbiological Monitoring and Bioremediation of Soils, All-Russia Research Institute for Agricultural Microbiology), or *Mesorhizobium ciceri* 2107 (*C. arietinum*) (obtained from the Russian Collection of Agricultural Microorganisms, All-Russia Research Institute for Agricultural Microbiology, Saint-Petersburg, Russia).

The plants were grown in sterile vermiculite wetted with nitrogen-free nutrient solution [31] in an MLR-352H growth chamber (Sanyo Electric Co., Ltd., Moriguchi, Japan) under controlled conditions: light/dark cycle, 16/8 h; temperature, 21 °C; humidity, 75%; and illumination, 280 µmol photons m^−2^ s^−1^). The *V. sativa* and *G. orientalis* nodules were harvested on the 14th day after inoculation, whereas the *C. arietinum* nodules were harvested on the 17th day.

### 2.2. Microscopy

#### 2.2.1. Scanning Electron Microscopy

For scanning electron microscopy, nodules were fixed in 2.5% glutaraldehyde (Sigma-Aldrich, St Louis, MO, USA) in 0.06 M phosphate buffer (pH 7.2). The nodules were dehydrated with a graded ethanol series and dried with a Leica EM CPD300 critical-point dryer (Leica Microsystems, Vienna, Austria). The specimens were then mounted on stubs, coated with 10 nm gold by a Leica EM SCD500 high-vacuum sputter coater (Leica Microsystems), and observed using a Tescan MIRA3 LMU scanning electron microscope (Tescan, Brno, Czech Republic) at 9 kV.

#### 2.2.2. Transmission Electron Microscopy

The transmission electron microscopy protocol was previously described [32]. Briefly, nodules were fixed in 2.5% glutaraldehyde in 0.1 M phosphate buffer (pH 7.2), post-fixed in 2% osmium tetroxide, and embedded in Eponate 12 (Ted Pella Inc., Redding, CA, USA). Thereafter, 90–100 nm thick ultrathin sections were cut using a diamond knife (Diatome, Bienne, Switzerland) on a Leica EM UC7 ultramicrotome (Leica Microsystems) and collected on copper grids coated with 4% formvar and carbon. The sections were counterstained with 2% aqueous uranyl acetate followed by lead citrate. The nodule tissues were examined and photographed using a JEOL-1400 transmission electron microscope (JEOL Corporation, Tokyo, Japan) at 80 kV.

#### 2.2.3. Immunolocalization and Laser Scanning Confocal Microscopy

Visualization of tubulin microtubules was performed as previously described [22] with several modifications, which are required for certain species [33]. For each species, an optimal fixing solution composition was developed. *G. orientalis* nodules were fixed in the fixative solution (3% formaldehyde, 0.25% glutaraldehyde, 0.3% Tween-20, 0.3% Triton X-100, and 0.5% dimethyl sulfoxide) in 1/8 microtubule-stabilizing buffer (MTSB; 50 mM PIPES, 5 mM MgSO_4_·7H_2_O, and 5 mM EGTA, pH 6.9). *V. sativa* nodules were fixed in the fixative solution (3% formaldehyde, 0.25% glutaraldehyde, 0.3% Tween-20, 0.3% Triton X-100, and 10% dimethyl sulfoxide) in 1/6 MTSB. *C. arietinum* nodules were fixed in the fixative solution (3% formaldehyde, 0.25% glutaraldehyde, 0.3% Tween-20, 0.3% Triton X-100, and 10% dimethyl sulfoxide) in 1/8 MTSB. Nodule longitudinal sections were cut using a microtome with a vibrating blade HM650V (Microm, Walldorf, Germany). To detect the tubulin in the cells, an anti-tubulin mouse monoclonal IgG antibody (clone DM1A; Sigma-Aldrich) was used, followed by an anti-mouse goat IgG secondary antibody conjugated with Alexa Fluor 488 (Thermo Fisher Scientific, Waltham, MA, USA). To visualize the infection droplets and infection threads, rat monoclonal antibody against the infection thread matrix glycoprotein (diluted 1:100; MAC265) [34] was used, followed by anti-rat goat IgG secondary antibody conjugated with Alexa Fluor 546 (Thermo Fisher Scientific). To identify the nuclei and bacteria, the sections were stained with propidium iodide (0.5 µg mL^−1^) for 7 min. After washing, the sections were placed under coverslips in ProLong Diamond^®^ antifade reagent (Thermo Fisher Scientific). Sections were analyzed using an LSM 780 laser scanning confocal microscope and ZEN 2012 software (Zeiss, Oberkochen, Germany). AlexaFluor 488 was excited at 488 nm, and fluorescence emitted between 499 to 543 nm was collected; Alexa Fluor 546 was excited at 561 nm, and emitted fluorescence between 568 and 572 nm was collected; propidium iodide was excited at 561, and emitted fluorescence between 606 and 677 nm was collected.

### 2.3. Quantitative Analysis

Full z-stack confocal images of individual nodule cells with labeled microtubules were subjected to the Phansalkar thresholding method and denoised using the ImageJ [35] Remove Outliers median filter. Thereafter, the images were smoothed using Gaussian blur (sigma: 0.5) and skeletonized using the ImageJ Skeletonize3D plugin [36]; noise branches were pruned using a script provided by the plugin developer. The skeletonized images were analyzed using the AnalyzeSkeleton plugin [36]. The result tables were then processed using a custom R script and six features were calculated for each cell: (1) total number of branches, which is the number of all detected microtubules (Figure S1A); (2) total length of branches, which is the total length of all detected microtubules (Figure S1A); (3) mean straightness index of detected microtubules, which is the Euclidian distance between the starting and ending point of each branch divided by its full length (Figure S1A); (4) total number of junctions (Figure S1A); (5) degree of branching, which is the number of skeletons (sets of branches connected) with more than one branch divided by the total number of skeletons in the image (Figure S1A,B); and (6) mean number of junctions per skeleton, which is the average number of branching points across all skeletons in the cell (Figure S1A,B). Statistically significant differences in these features between different species were determined using Tukey’s range test.

### 2.4. Bacteroid Isolation

The nitrogen fixation zone was separated from the formaldehyde-fixed nodules (5 nodules for each species) and cut into pieces with a razor blade. Next, pieces of the nodules were stained with propidium iodide (as described above) and ground with a glass pestle in 100 µL Tris-buffered saline. The length of 50 bacteroids of each species was determined. Pairwise comparisons were conducted using Tukey’s range test.

### 2.5. Measurement of the Intensity of Antibody-Labeled Endoplasmic Microtubules

For intensity measurements, we used the maximum intensity projections of confocal images of individual nodule nitrogen-fixing cells (5 cells for each species) obtained using identical settings. For each cell, the intensity was measured in four regions of interest (ROIs), which involved sampling one-third of the whole-cell along each of the four axes. A higher intensity of labeled microtubules may reflect a higher density of microtubule bundles. Pairwise comparisons of ROI intensities between different species were conducted using Tukey’s range test.

## 3. Results

### 3.1. Symbiosome Distribution in Infected Cells and Bacteroid Morphology

**Figure 1:**
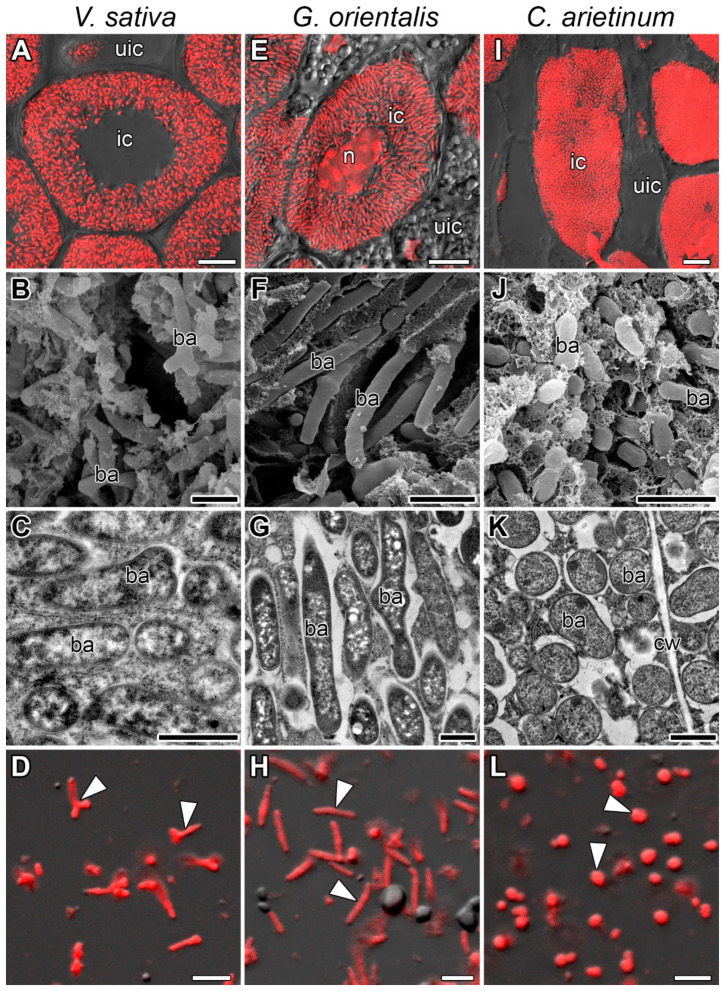
Infected cells and bacteroids. (**A**–**D**) *Vicia sativa* L., (**E**–**H**) *Galega orientalis* Lam, and (**I**–**L**) *Cicer arietinum* L. (**A**,**E**,**I**,) Symbiosome arrangement in infected cells of the nitrogen-fixation zone. (**B**–**L**) Bacteroid morphology based on (**B**,**F**,**J**) scanning electron microscopy, (**C**,**G**,**K**) transmission electron microscopy, and (**D**,**H**,**L**) laser scanning confocal microscopy. (**A**,**E**,**I**,**D**,**H**,**L**) Merged images of differential interference contrast and red channel (DNA staining with propidium iodide (nuclei and bacteria)). ba, bacteroid; cw, cell wall; n, nucleus; ic, infected cell; uic, uninfected cell; arrowheads indicate bacteroids. Bars: (**A**,**E**,**I**) 10 µm, (**B**,**F**,**J**) 2 µm, (**C**,**G**,**K**) 1 µm, and (**D**,**H**,**L**) 5 µm.

**Figure 2:**
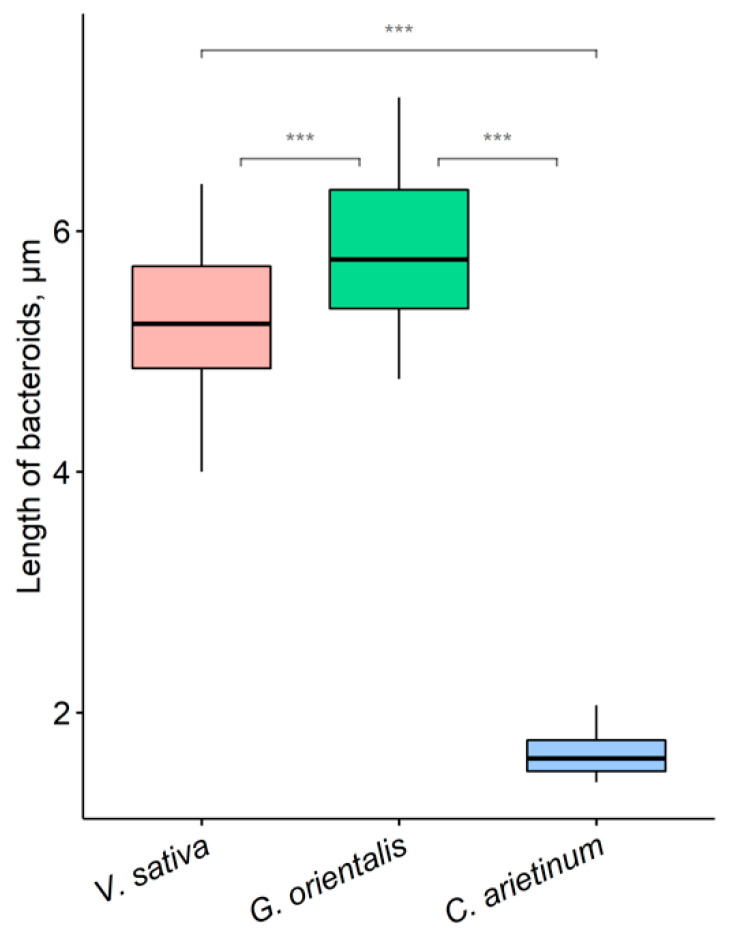
Length of bacteroids in nitrogen-fixing cells of *Vicia sativa* L., *Galega orientalis* Lam, and *Cicer arietinum* L. nodules. Pairwise comparisons were conducted using Tukey’s range test, *** *p* < 0.001; *n* = 50.

Symbiosomes of *V. sativa*, *G. orientalis*, and *C. arietinum* differed regarding their distribution in infected cells (Figure 1A,E,I). In infected *V. sativa* (Figure 1A) and *C. arietinum* (Figure 1I) cells, symbiosomes were randomly oriented, whereas in infected *G. orientalis* cells, they were mainly oriented perpendicular to the cell surface and parallel to each other (Figure 1E). Analysis of the ultrastructural organization of the nodules and confocal images of isolated bacteroids demonstrated that bacteroids of *G. orientalis* and *V. sativa* belong to the E morphotype (Figure 1B–D,F–H), whereas bacteroids of *C. arietinum* belong to the S morphotype (Figure 1J–L). However, the *G. orientalis* bacteroids are barely branched (ENB morphotype) (Figure 1F–H), whereas the *V. sativa* bacteroids are characterized by intensive branching (EB morphotype) (Figure 1B–D). The bacteroids of the studied species vary significantly in size. The smallest was observed in *C. arietinum*, *V. sativa* bacteroids were of intermediate size, and the largest was *G. orientalis* bacteroids, reaching 6–7 µm in length (Figure 2).

### 3.2. Microtubule Organization in Meristematic Cells

**Figure 3:**
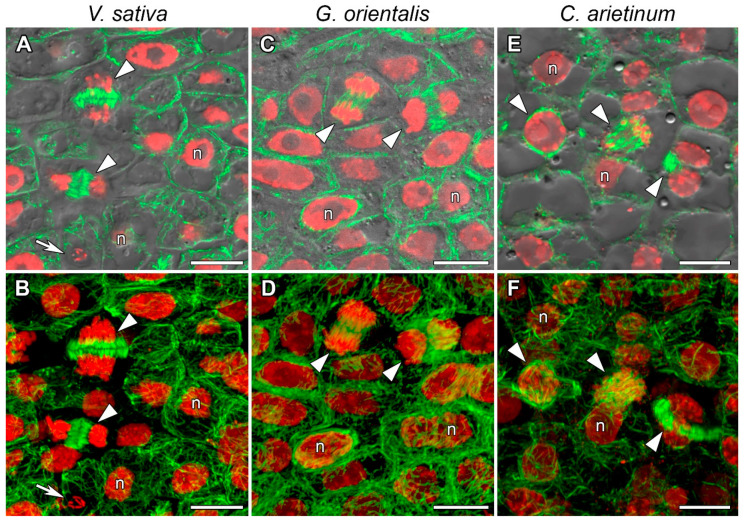
Microtubule organization in meristematic cells. (**A**,**B**) *Vicia sativa* L., (**C**,**D**) *Galega orientalis* Lam, and (**E**,**F**) *Cicer arietinum* L. Confocal laser scanning microscopy of 50 µm longitudinal vibratome sections. (**A**–**F**) Immunolocalization of tubulin (microtubules), green channel; DNA staining with propidium iodide (nuclei and bacteria), red channel. (**A**,**C**,**E**) Merged images of a single optical section of differential interference contrast and maximum intensity projection of optical sections in green and red channels. (**B**,**D**,**F**) Maximum intensity projections of (**B**) 50, (**D**) 40, and (**F**) 45 optical sections in green and red channels. n, nucleus; arrowheads indicate mitotic figures, arrows indicate infection threads. Bars, 10 µm.

The cells of the meristem are characterized by small size and having a centrally located nucleus and small vacuoles. In nodules of *V. sativa* (Figure 3A,B, Video S1), *G. orientalis* (Figure 3C,D, Video S2), and *C. arietinum* (Figure 3E,F, Video S3) meristematic cells, the cortical microtubules were randomly organized and crossed at different angles, forming a dense network. Perinuclear microtubules were arranged in a dense network around the nucleus, while some of them connected the nucleus to the plasma membrane of the cell (Figure 3, Videos S1–S3). Microtubules formed different mitotic arrays like preprophase bands and mitotic spindles (Figure 3, Videos S1–S3).

### 3.3. Microtubule Organization in Infected Cells in the Infection Zone

**Figure 4:**
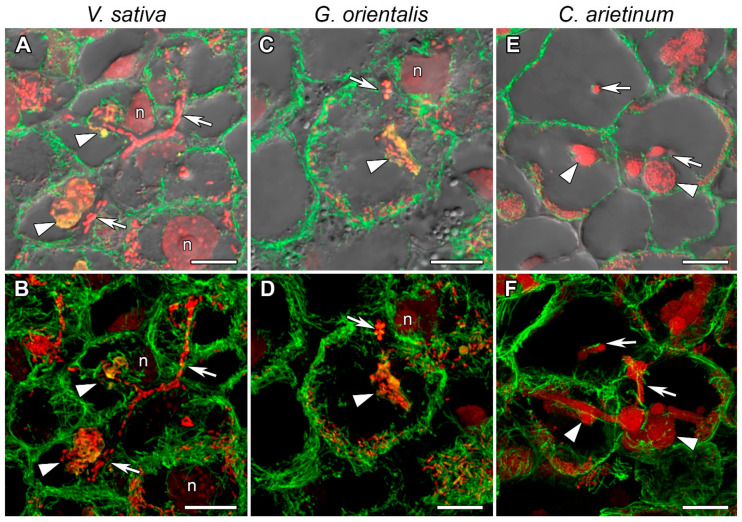
Endoplasmic microtubule organization in infected cells of infection zone. (**A**,**B**) *Vicia sativa* L., (**C**,**D**) *Galega orientalis* Lam, and (**E**,**F**) *Cicer arietinum* L. Confocal laser scanning microscopy of 50 µm longitudinal vibratome sections. (**A**–**F**) Immunolocalization of tubulin (microtubules), green channel; DNA staining with propidium iodide (nuclei and bacteria), red channel. (**A**–**D**) Immunolocalization of MAC265 (infection droplets), yellow channel. (**A**,**C**) Merged images of a single optical section of differential interference contrast and maximum intensity projection of optical sections in green, yellow and red channels; (**E**) Merged image of a single optical section of differential interference contrast and maximum intensity projection of optical sections in green and red channels. (**B**,**D**,**F**) Maximum intensity projections of (**B**,**D**) 40 optical sections in green, yellow and red channels and (**F**) 45 optical sections in green and red channels. n, nucleus; arrowheads indicate infection droplets; arrows indicate infection threads. Bars, 10 µm.

In nodules of *V. sativa* (Figure 4A,B), *G. orientalis* (Figure 4C,D), and *C. arietinum* (Figure 4E,F) in the infection zone, some of the cells were infected with rhizobia released from infection droplets, and the infected cells underwent differentiation and significantly increased in size. Other cells remained uninfected. In these infected nodule cells in the infection zone, a similar cortical and endoplasmic microtubule organization was observed in all studied species. The cortical microtubules were located at different angles and formed an irregular pattern (Video S4). Thick bundles of endoplasmic microtubules passed along the infection threads and formed a network around the infection droplets and nuclei (Figure 4, Video S4). The cells were filled with symbiosomes and the endoplasmic microtubules were located among the symbiosomes (Figure 4).

### 3.4. Microtubule Organization in Uninfected and Colonized Cells

**Figure 5:**
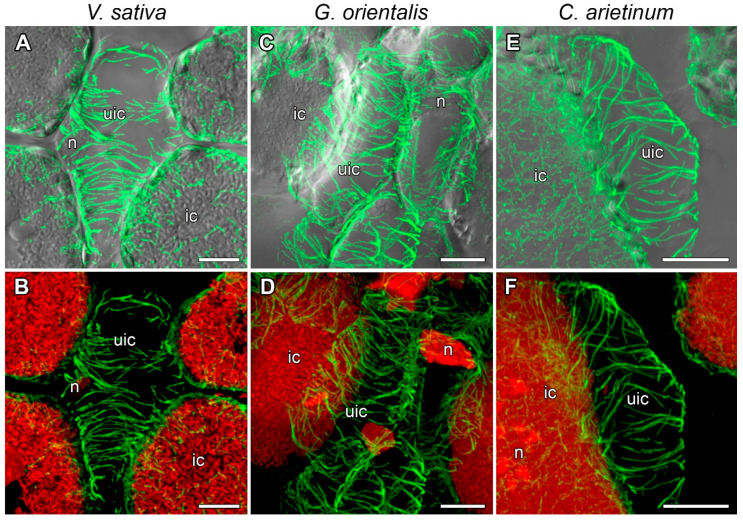
Cortical microtubule organization in uninfected cells. (**A**,**B**) *Vicia sativa* L., (**C**,**D**) *Galega orientalis* Lam, and (**E**,**F**) *Cicer arietinum* L. Confocal laser scanning microscopy of 50 µm longitudinal vibratome sections. (**A**–**F**) Immunolocalization of tubulin (microtubules), green channel; DNA staining with propidium iodide (nuclei and bacteria), red channel. (**A**,**C**,**E**) Merged images of a single optical section of differential interference contrast and maximum intensity projection of optical sections in green channel. (**B**,**D**,**F**) Maximum intensity projections of (**B**) 30, (**D**) 70, and (**F**) 50 optical sections in green and red channels. n, nucleus; ic, infected cell; uic, uninfected cell. Bars, 10 µm.

In nodules of *V. sativa* (Figure 5A,B), *G. orientalis* (Figure 5C,D), and *C. arietinum* (Figure 5E,F) in uninfected cells, the cortical microtubules formed a regular pattern. They were located parallel to each other and perpendicular to the longitudinal axis of the cell in all studied species (Figure 5; Video S5). Endoplasmic microtubules could not be identified. A similar pattern persisted in colonized cells that contained infection threads and droplets without bacterial release (Figure S2). Endoplasmic microtubules were characterized by thick bundles extending along the infection threads (Figure S2).

### 3.5. Microtubule Organization in Infected Cells in the Nitrogen Fixation Zone

**Figure 6:**
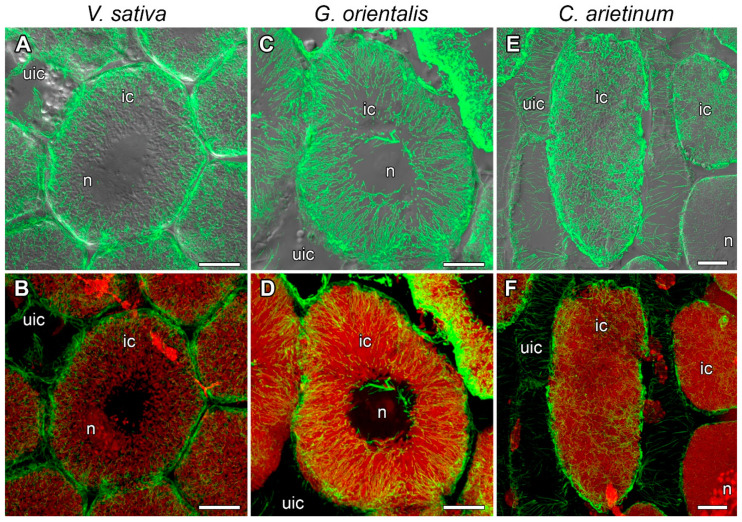
Endoplasmic microtubule organization in infected cells of nitrogen fixation zone. (**A**,**B**) *Vicia sativa* L., (**C**,**D**) *Galega orientalis* Lam, and (**E**,**F**) *Cicer arietinum* L. Confocal laser scanning microscopy of 50 µm longitudinal vibratome sections. (**A**–**F**) Immunolocalization of tubulin (microtubules), green channel; DNA staining with propidium iodide (nuclei and bacteria), red channel. (**A**,**C**,**E**) Merged images of a single optical section of differential interference contrast and maximum intensity projection of optical sections in green channel. (**B**,**D**,**F**) Maximum intensity projections of 40 optical sections in green and red channels. n, nucleus; ic, infected cell; uic, uninfected cell. Bars, 10 µm.

In the *V. sativa* (Figure S3A), *G. orientalis* (Figure S3B), and *C. arietinum* (Figure S3C) nodule cells, cortical microtubules formed an irregular pattern. In the *G. orientalis* nodules, endoplasmic microtubules formed a dense network (Video S6) in which they were oriented radially and parallel to each other between differentiated symbiosomes, which led to regular orientation of the symbiosomes (Figure 6C,D). In the *V. sativa* nodule cells, the microtubules were, to some extent, oriented radially but mainly irregularly (Figure 6A,B, Video S7) and symbiosomes were randomly distributed in the cell (Figure 6A,B). Endoplasmic microtubules in the *C. arietinum* infected cells formed a dense network (Video S8) among symbiosomes, which were randomly distributed in the cell (Figure 6B,F).

### 3.6. Quantitative Analysis

**Figure 7:**
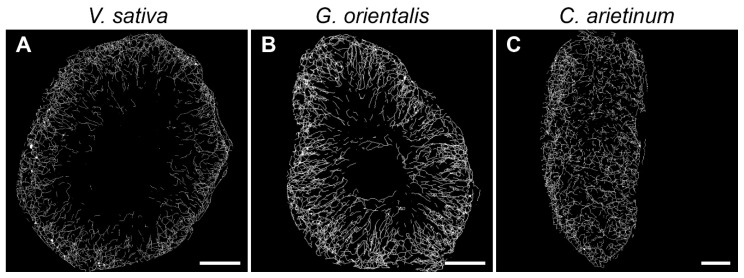
Skeletonized microtubules of nitrogen-fixing cells of symbiotic nodules. (**A**) *Vicia sativa* L., (**B**) *Galega orientalis* Lam, and (**C**) *Cicer arietinum* L. Bars, 10 µm.

**Figure 8:**
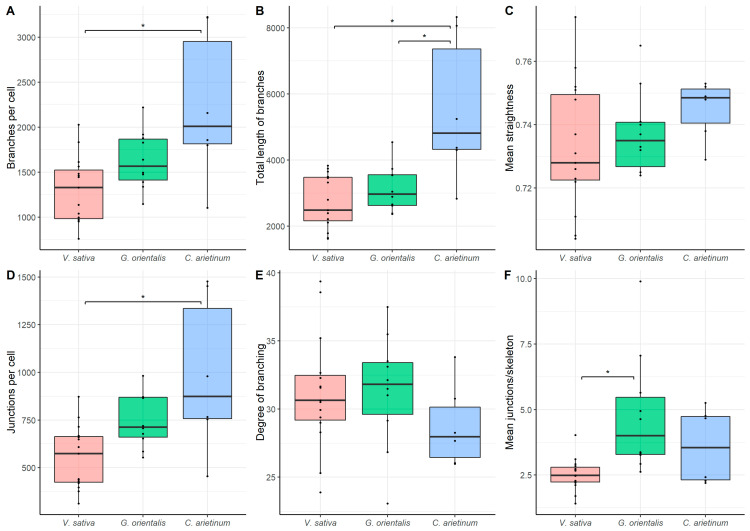
Quantitative analysis of tubulin microtubules in nitrogen-fixing cells of *Vicia sativa* L. (*n* = 15), *Galega orientalis* Lam (*n* = 10), and *Cicer arietinum* L. (*n* = 6) nodules. (**A**) Total number of branches per cell (number of all detected microtubules). (**B**) Total length of branches per cell (the total length of all detected microtubules per cell). (**C**) Mean straightness index of detected microtubules per cell (the Euclidian distance between the starting and ending point of each branch divided by its full length). (**D**) Total number of junctions per cell. (**E**) Degree of branching per cell (number of skeletons (sets of branches, connected together) with more than one branch divided by the total number of skeletons in the image). (**F**) Mean number of junctions per skeleton (average number of branching points across all skeletons in the cell). Pairwise comparisons were conducted using Tukey’s range test, * *p* < 0.05.

Using Skeletonize3D software, images of the microtubule were skeletonized (Figure 7). The following parameters were explored: (1) total number of microtubules, (2) total length of microtubules, (3) mean straightness index of detected microtubules, (4) total number of junctions, (5) degree of branching, and (6) mean number of junctions per skeleton (Figure S1). The quantitative analysis of endoplasmic microtubule organization revealed that infected *C. arietinum* cells had the highest total number of microtubules, length of microtubules, and number of junctions per cell, and they significantly differed in these parameters from *V. sativa* cells (Figure 8A,B,D). Infected *C. arietinum* and *G. orientalis* cells significantly differed in the total length of microtubules (Figure 8B). Infected *G. orientalis* and *V. sativa* cells significantly differed in the mean number of junctions per skeleton (Figure 8F). The mean straightness index of detected microtubules and the degree of branching were similar among the infected cells of all studied species (Figure 8C,E).

### 3.7. Mean Intensity of Antibody-Labeled Endoplasmic Microtubules

Measurements of the mean intensity of antibody-labeled endoplasmic microtubules in confocal images of nitrogen-fixing cells of *V. sativa*, *G. orientalis*, and *C. arietinum* revealed that *G. orientalis* significantly differs from the two other species regarding this parameter (Table 1).

## 4. Discussion

In this study, the tubulin cytoskeleton organization in the cells of indeterminate nodules in three legume species was assessed. These species belong to the Vicioid subclade of the inverted repeat-lacking clade (IRLC) [37], which includes species with terminated differentiation of bacteroids due to the activity of NCR peptides [10]. Previously, the tubulin cytoskeleton organization in indeterminate nodules of two legume species belonging to IRLC clade was studied, and both similarities and species-specific features were revealed [22].

The analyzed species differ in terms of bacteroid morphotypes, with the tested rhizobia strains forming the ENB morphotype in *G. orientalis* nodules, the EB morphotype in *V. sativa* nodules, and the S morphotype in *C. arietinum* nodules (Figure 1). In general, these morphotypes correspond well to morphotypes previously described for *C. arietinum* [38], but not for *G. orientalis* [30]. The longest bacteroids are in *G. orientalis* nodules, the shortest in *C. arietinum* nodules, and in *V. sativa*, they are of intermediate size (Figure 2).

The tubulin cytoskeleton organization in the meristematic cells (Figure 3, Videos S1–S3) is the same as those in previously studied indeterminate nodules of *P. sativum* and *M. truncatula* [22] and similar to that in meristematic root cells [39,40]. In the cells in the infection zone, endoplasmic microtubules are involved in the growth of infection threads and droplets and in the distribution of bacteria released into the cell (Figure 4, Video S4), as previously observed in both *P. sativum* and *M. truncatula* [22]. Cortical microtubules in uninfected and colonized cells formed a regular pattern (microtubules were located parallel to each other and perpendicular to the longitudinal axis of the cell) (Figure 5 and Figure S2; Video S5). A similar pattern was observed in *P. sativum* and *M. truncatula* nodule cells [22]. The identified organization is likely universal for uninfected cells of indeterminate nodules, which allows them to maintain their anisotropic growth. It is known that a regular cortical microtubule pattern allows for anisotropic growth of plant cells [41,42,43]. This pattern is common for root cells passing from the transition zone to the elongation zone [40,44]. Additionally, there are no differences among the analyzed species in the cortical microtubule pattern in infected nodule cells in the nitrogen fixation zone (Figure S3). Cortical microtubules in the *G. orientalis* and *V. sativa* nodule cells form an irregular pattern to allow isodiametric cell growth. Infected cells of these species are spherical or ellipsoid. The cortical microtubules in *C. arietinum* also form an irregular pattern, although the mature infected cells are more elongated than the cells of the two other species. It was shown that in root cells, both auxin and cytokinin are involved in the reorientation of cortical microtubules. The involvement of auxin has been shown in the rapid transition of cortical microtubules from transverse to longitudinal organization in root and hypocotyl cells of *Arabidopsis thaliana* (L.) Heynh. [45,46]. Recently, it was demonstrated that cytokinins control the rearrangement of cortical microtubules from transverse orientation in the transition and elongation zones to oblique orientation in the differentiation zone in the epidermal cells of *A. thaliana* root [47]. Intriguingly, the localization of auxin in the symbiotic nodules of *L. japonicus* [48] and *P. sativum* [49] is limited to the meristem and vascular tissues. Active accumulation of cytokinins was observed in *P. sativum* nodules not only in the meristem, but also in the infection zone and the distal part of the nitrogen fixation zone [49]. Moreover, in the *P. sativum* mutant *sym33-3* [50], which is characterized by an ordered pattern of cortical microtubules in colonized cells [22], the localization of cytokinins is mainly limited to the meristem [49]. Nevertheless, additional studies are needed to confirm the possible involvement of cytokinins in the reorientation of cortical microtubules not only in the root, but also in the symbiotic nodule.

Previously, it was shown that in the nitrogen-fixing cells of *M. truncatula* and *P. sativum* nodules, the endoplasmic microtubule organization is associated with the arrangement of intracellular symbiosomes [22]. The endoplasmic microtubule organization in infected *G. orientalis* nodule cells in the nitrogen fixation zone is similar to that in corresponding *M. truncatula* cells, i.e., endoplasmic microtubules are organized radially, perpendicular to the cell wall, and pass along the symbiosomes (Figure 6C,D, Video S6). In both species, the bacteroids belong to the ENB morphotype; they are also the longest among the studied species. Previously, it was demonstrated that in *P. sativum*, symbiosomes (with EB morphotype bacteroids) are arranged randomly, and endoplasmic microtubules form a dense network between them. A similar endoplasmic microtubule pattern was observed in infected *V. sativa* (Figure 6A,B, Video S7) and *C. arietinum* (Figure 6E,F, Video S8) nodule cells. However, in infected *V. sativa* cells, microtubules demonstrated a tendency toward the radial organization. Interestingly, the bacteroids of *V. sativa* are longer than bacteroids of *P. sativum* and not much shorter than the bacteroids of *G. orientalis* or *M. truncatula*. Additionally, the bacteroids of *V. sativa*, like bacteroids of *P. sativum*, belong to the EB morphotype. Nevertheless, in both infected *V. sativa* and *C. arietinum* nodule cells, symbiosomes with EB and S morphotype bacteroids, respectively, were arranged irregularly. There was no difference in the mean intensity of antibody-labeled endoplasmic microtubules between *V. sativa* and *C. arietinum* nodules, but both species significantly differed from *G. orientalis* (Table 1). This confirmed the different (i.e., parallel and radial) endoplasmic microtubule patterns in *G. orientalis* nodules. Quantitative analysis revealed significant differences in the endoplasmic microtubule patterns, particularly between *C. arietinum* and *V. sativa* (Figure 8). In *C. arietinum* nodule cells, we observed a denser endoplasmic microtubule network, which arranged the numerous symbiosomes with small bacteroids. Thus, it can be assumed that the endoplasmic microtubule pattern in infected cells of indeterminate nodules is associated with the bacteroid morphology (both shape and size), and this pattern determines the symbiosome distribution in the infected cell.

By placing a large number of symbiosomes in an infected cell, the plant solves a complex topological problem, i.e., filling its volume with the maximum number of elements of a certain shape and size. It is likely that for elongated *G. orientalis* and *M. truncatula* bacteroids, such an optimal arrangement is a regular (radial) one, whereas for pleiomorphic *V. sativa* and *P. sativum* bacteroids, it is irregular (to some extent, the orientation of bacteroids in these species resembles the arrangement of Tetris elements). Small spherical *C. arietinum* bacteroids probably also do not require ordered packing to effectively fill the volume of an infected cell. Accordingly, endoplasmic microtubules organize into patterns capable of efficiently filling an infected cell with symbiosomes. The development of topological models can be extremely helpful in addressing these issues.

Notably, along with the tubulin cytoskeleton, a role for the actin cytoskeleton has been suggested in the distribution of symbiosomes. For example, for *G. max* nodules, it was shown that the actin array is a honeycomb pattern that is involved in symbiosome distribution, whereas microtubules are involved in the distribution of different organelles on the cell periphery but not symbiosomes [19]. In *M. truncatula* nodules, actin was involved in the accommodation of juvenile symbiosomes in young infected cells, forming a network around symbiosomes [51,52].

## 5. Conclusions

The results of this study confirm the universality of the tubulin microtubule organization in many nodule cells across legume species and the participation of microtubules in the infection process in the three studied species. The microtubule organization in the meristematic cells, infected cells in the infection zone, and uninfected cells in nodules are similar among the three legume species with indeterminate nodules. The species-specific difference involves the endoplasmic microtubule organization in nitrogen-fixing cells, which depends on bacteroid morphology (shape and size) and determines the intracellular symbiosome distribution. To date, three types of network organization of endoplasmic microtubules involved in the symbiosome distribution in the cell have been identified. The first pattern, which is exhibited by *G. orientalis* and *M. truncatula* cells, involves endoplasmic microtubules that are located radially and parallel to each other, and symbiosomes that are similarly located. The second pattern, which is typical for *V. sativa* and *P. sativum* cells, involves endoplasmic microtubules forming an irregular pattern and disordered symbiosomes. Finally, in the third pattern, which is characteristic of *C. arietinum* cells, the endoplasmic microtubules are located irregularly, and they form a dense network, which is necessary for the ordered arrangement of numerous intracellular symbiosomes with small bacteroids. Further studies of microtubule patterns in a variety of species that form indeterminate nodules and determinate nodules will contribute to a deeper understanding of the role of the tubulin cytoskeleton in the development of symbiotic nodules.

## Figures and Tables

**Table 1:** Mean intensity of antibody-labeled endoplasmic microtubules in confocal images of nitrogen-fixing cells of *V. sativa*, *G. orientalis*, and *C. arietinum*. Pairwise comparisons were conducted using Tukey’s range test. ^a^ significant difference vs. *V. sativa*, ^b^ significant difference vs. *G. orientalis*; *n* = 5.

*Vicia sativa*	*Galega orientalis*	*Cicer arietinum*
133.67 ± 9.697 ^b^	247.31 ± 24.262 ^a^	130.9 ± 24.015 ^b^

## Data Availability

The data presented in this study are available in article and supplementary material.

## Supplementary Materials

The following are available online at https://www.mdpi.com/article/10.3390/cells10051012/s1, Video S1: Organization of microtubules in the meristematic cells of *Vicia sativa* L. nodules; Video S2: Organization of microtubules in the meristematic cells of *Galega orientalis* Lam nodules; Video S3: Organization of microtubules in the meristematic cells of *Cicer arietinum* L. nodules; Video S4: Organization of microtubules around infection structures in cells of the infection zone in *Cicer arietinum* L. nodules; Video S5: Organization of cortical microtubules in the uninfected cells of *Galega orientalis* Lam nodules; Video S6: Organization of microtubules in the infected cells of the nitrogen fixation zone of *Galega orientalis* Lam nodules; Video S7: Organization of microtubules in the infected cells of the nitrogen fixation zone of *Vicia sativa* L. nodules; Video S8: Organization of microtubules in the infected cells of the nitrogen fixation zone of *Cicer arietinum* L. nodules; Figure S1: Graphical representation of terms used in the quantitative analysis of the microtubule cytoskeleton; Figure S2: Organization of the cortical microtubules in the infected cells of the nitrogen fixation zone; Figure S3: Organization of microtubules in colonized cells.
