# Supplementary material for: General Patterns and Species-Specific Differences in the Organization of the Tubulin Cytoskeleton in Indeterminate Nodules of Three Legumes
Source: Cells. 2021 Apr 25;10(5):1012. doi: 10.3390/cells10051012 (PMC8146709; doi:10.3390/cells10051012)
Supplement: Supplementary file 1 [file cells-10-01012-s001.zip › cells-1146942/Supplementary_materials.pdf]

Supplementary materials

General patterns and species-specific differences in the organization of the tubulin cytoskeleton in indeterminate nodules of three legumes

Anna B. Kitaeva, Artemii P. Gorshkov, Evgenii A. Kirichek, Pyotr G. Kusakin, Anna V. Tsyganova and Viktor E. Tsyganov*

All-Russia Research Institute for Agricultural Microbiology, Laboratory of Molecular and Cellular Biology, Podbelsky chaussee 3, 196608, Pushkin 8, Saint-Petersburg, Russia; anykitaeva@gmail.com (A.B.K.); artemius1993@yandex.ru (A.P.G.); jenykir@rambler.ru (E.A.K.); kussakin@gmail.com (P.G.K.); avtsyganova@arriam.ru (A.V.T.)

* Correspondence: vetsyganov@arriam.ru

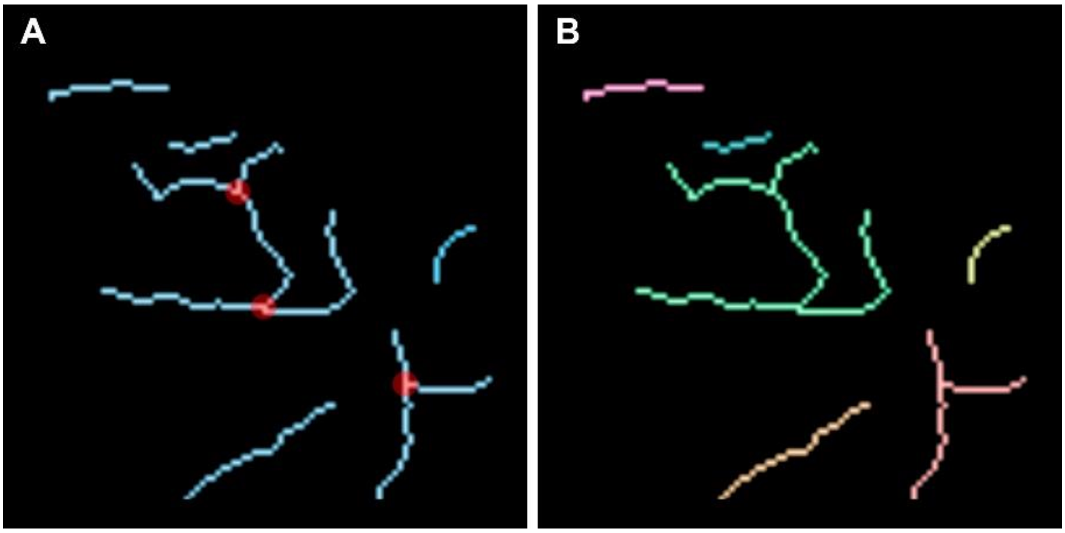

Figure S1. Graphical representation of terms used in quantitative analysis of microtubule cytoskeleton. Fragment of the 3d-reconstruction of skeletonized confocal image of microtubule cytoskeleton. (A) Junction points indicated by red circles; individual branches colored in blue. (B) Skeletons, each skeleton is labeled with a different color.

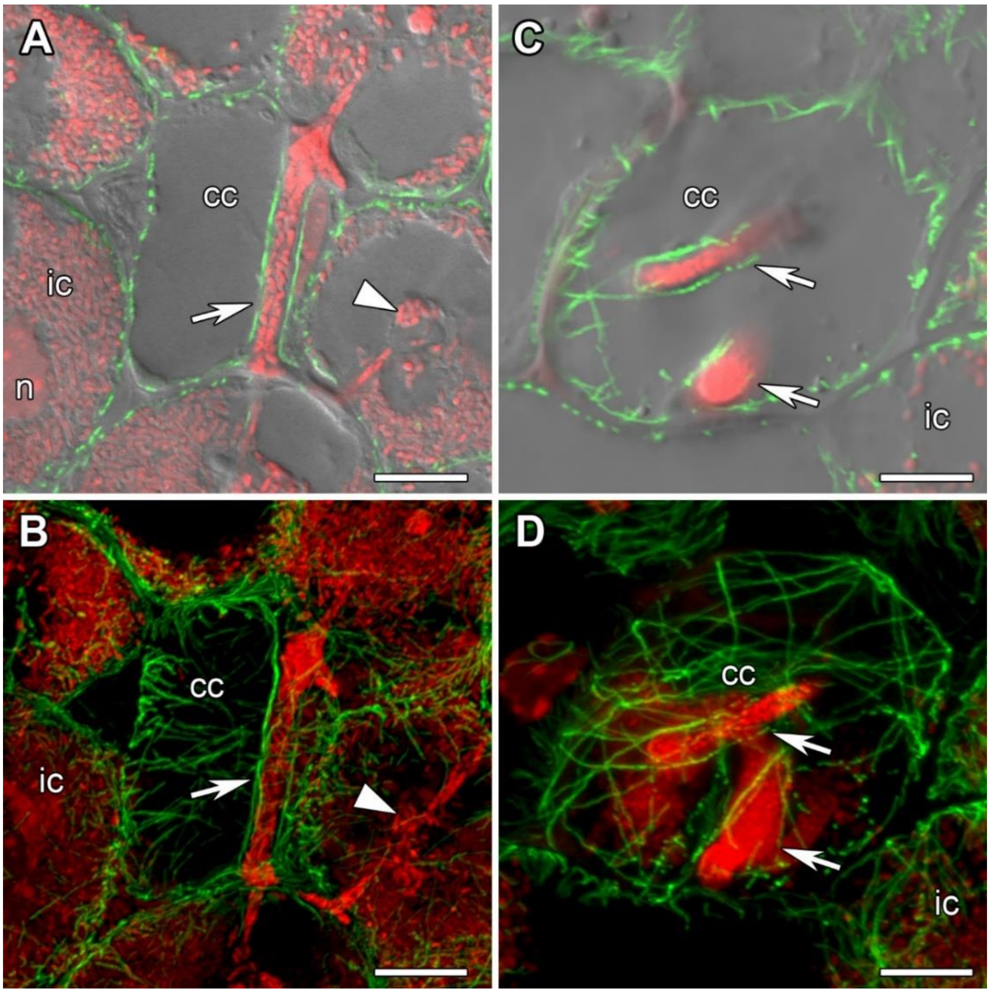

Figure S2. Organization of microtubules in colonized cells

(A, B) *Vicia sativa* L., (C, D) *Cicer arietinum* L. Confocal laser scanning microscopy of longitudinal 50 μm vibratome sections. (A, B) Immunolocalization of tubulin (microtubules), green channel; DNA staining with propidium iodide (nuclei and bacteria), red channel. (A, C) merge of a single optical section of differential interference contrast and maximum intensity projection of optical sections in green and red channels. (B, D) maximum intensity projections of 50 optical sections in green and red channels. n, nucleus; ic, infected cell; cc, colonized cell; arrowheads indicate infection droplets; arrows indicate infection threads. Bars, 10 μm.

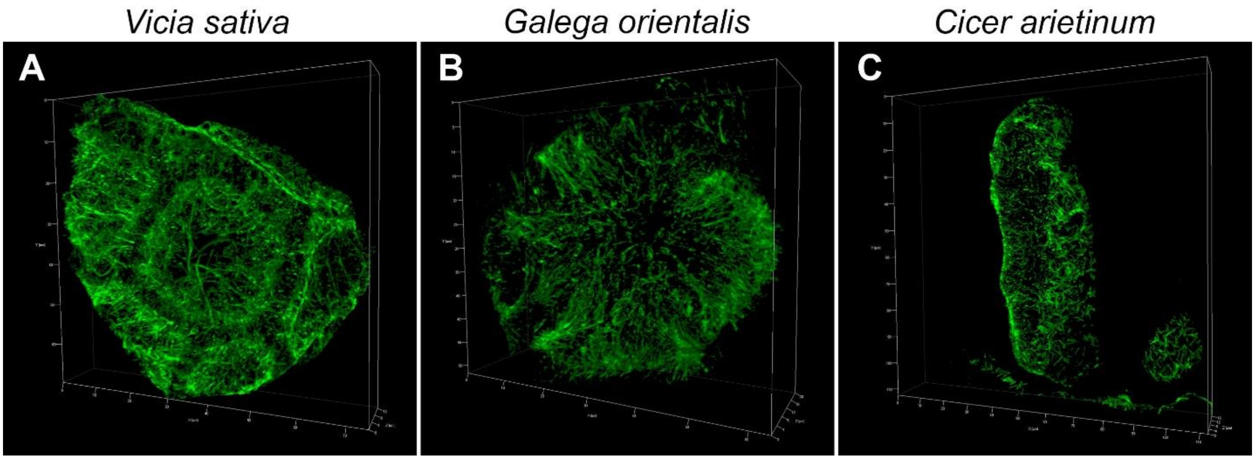

Figure S3. Organization of cortical microtubules in infected cells of nitrogen fixation zone 3D-reconstructions of z-stacks of 50 optical sections. Microtubules are green.
